# Implementation and evaluation of a communication coaching program: a CFIR-Informed qualitative analysis mapped onto a logic model

**DOI:** 10.1186/s12909-025-07188-6

**Published:** 2025-04-25

**Authors:** Rachel M. Jensen, Marzena Sasnal, Uyen T. Mai, James R. Korndorffer, Rebecca K. Miller-Kuhlmann, Arden M. Morris, Aussama K. Nassar, Carl A. Gold

**Affiliations:** 1 Department of Surgery, Stanford University School of Medicine, Stanford, CA USA; 2 Stanford-Surgery Policy Improvement Research and Education Center (S-SPIRE), Department of Surgery, Stanford University School of Medicine, Stanford, CA USA; 3 Center for Research On Education Outcomes, Stanford University, Stanford, CA USA; 4 Department of Neurology and Neurological Sciences, Stanford University School of Medicine, Stanford, CA USA

**Keywords:** Graduate Medical Education (GME), Communication Coaching, Program Evaluation, Program Implementation, The Consolidated Framework of Implementation Research, Logic Model, Qualitative Interview Study

## Abstract

**Background:**

Coaching programs in graduate medical education have the potential to impact trainee development across multiple core competencies but require rigorous program evaluation to ensure effectiveness. We sought to qualitatively evaluate the implementation of a multi-departmental, faculty-led communication coaching program using a logic model framework.

**Methods:**

Study participants were selected from four key stakeholder groups: resident coachees, faculty coaches, medical education leaders, and programmatic sponsors. 30–45 min semi-structured interviews were conducted via Zoom, transcribed, and de-identified for the analysis. Interviews captured stakeholders' perspectives on physicians' communication training needs, stakeholders perceived and actual roles, stakeholders’ involvement in the program, factors influencing the implementation process, and strategies for programmatic improvement, sustainment, and spread. The Consolidated Framework of Implementation Research (CFIR) guided the codebook development and data analysis. A combined inductive/deductive approach was used to develop a 20-item codebook, followed by a team-based thematic analysis. A strong intercoder agreement (Cohen’s kappa coefficient *κ* = 0.83) ensured coding consistency. The emerging themes were then mapped onto four domains of a logic model: Context, Inputs and Outputs, Outcomes, and Evaluation.

**Results:**

35 interviews were conducted between November 2021 and April 2022 with representation from all stakeholder groups, including 10 resident coachees (who received coaching), 10 faculty coaches (who served as coaches and underwent coaching-specific faculty development), 9 medical education leaders (who designed and implemented program), and programmatic sponsors (who provided financial support). We mapped 8 emergent themes onto the critical domains of a logic model for program evaluation. For the domain of Context, themes included (1) gap in communication education and (2) patient-centeredness. For the domain of Inputs/Outputs, themes included (1) investment in the program and (2) perceived program value. For the domain of Outcomes, themes included (1) learning-focused outcomes and (2) patient-related outcomes. For the domain of Evaluation, themes included (1) defining success and (2) challenges with evaluation.

**Conclusions:**

Mapping CFIR-informed themes onto a logic model for program evaluation presents a novel strategy for integrating program implementation and evaluation, both of which are essential to effective educational programming. These findings can be used to guide future programmatic modifications to better meet the needs of key stakeholders.

## Background

Coaching programs at the graduate medical education (GME) level have been implemented to address the specific needs of trainees [1–3]. Coaching, which is distinct from mentorship or advising, involves a true partnership between a coach and a coachee [4]. A coaching framework is learner-centered and helps the coachee identify opportunities for improvement through facilitated self-reflection and goal setting [5, 6]. Coaching programs have enormous potential to impact resident growth across multiple core competencies, including practice-based learning and improvement, professionalism, and interpersonal skills and communication [1, 3]. Proficiency-focused efforts are critical to help learners progress along milestones in the current era of competency-based medical education. The Stanford Neurology and General Surgery Resident Communication Coaching Programs launched in 2020 and have engaged hundreds of residents in longitudinal coaching relationships focused on interpersonal skills and communication [7, 8].

Despite the strong potential of coaching programs, it is unclear whether coaching implementation efforts are effectively meeting the needs of stakeholders and inducing true change in learners [9]. Effective programmatic change requires deliberate implementation and rigorous evaluation. There is an opportunity cost associated with any new implementation effort in GME; the time and effort that a coach or coachee puts into one endeavor inevitably means less time and effort elsewhere. For this reason, programs must make difficult choices and evolve to effectively meet stakeholders’ needs [10, 11]. Intentional selection of implementation and evaluation frameworks may be critical to programmatic effectiveness. One common framework for programmatic implementation and evaluation is a logic model, which helps to balance community needs, program inputs and outputs, outcome measurements, and evaluation strategies [12, 13]. A logic model can be used in an a priori fashion to assist with program design and implementation or in a post hoc setting for program evaluation.

To evaluate outcomes related to a coaching intervention, programs often rely on survey-based quasi-experimental study designs; however, this approach may provide a limited view of a program’s impact and does not allow for exploration beyond the survey’s measured constructs [9]. Qualitative approaches, using individual interviews or focus groups, have the potential to provide a more robust program evaluation, especially for complex interventions with multiple stakeholders and moving parts. Therefore, we conducted a qualitative program evaluation of a faculty-led communication coaching program implemented at a single institution for Surgery and Neurology residents using semi-structured interviews with key program stakeholders. The goal of this novel strategy for integrating program implementation and evaluation is to describe a new framing that may benefit future educators and medical education researchers by bringing to the foreground concepts that are often missed in evaluation of programs.

## Methods

### Program Implementation

**Fig. 1:**
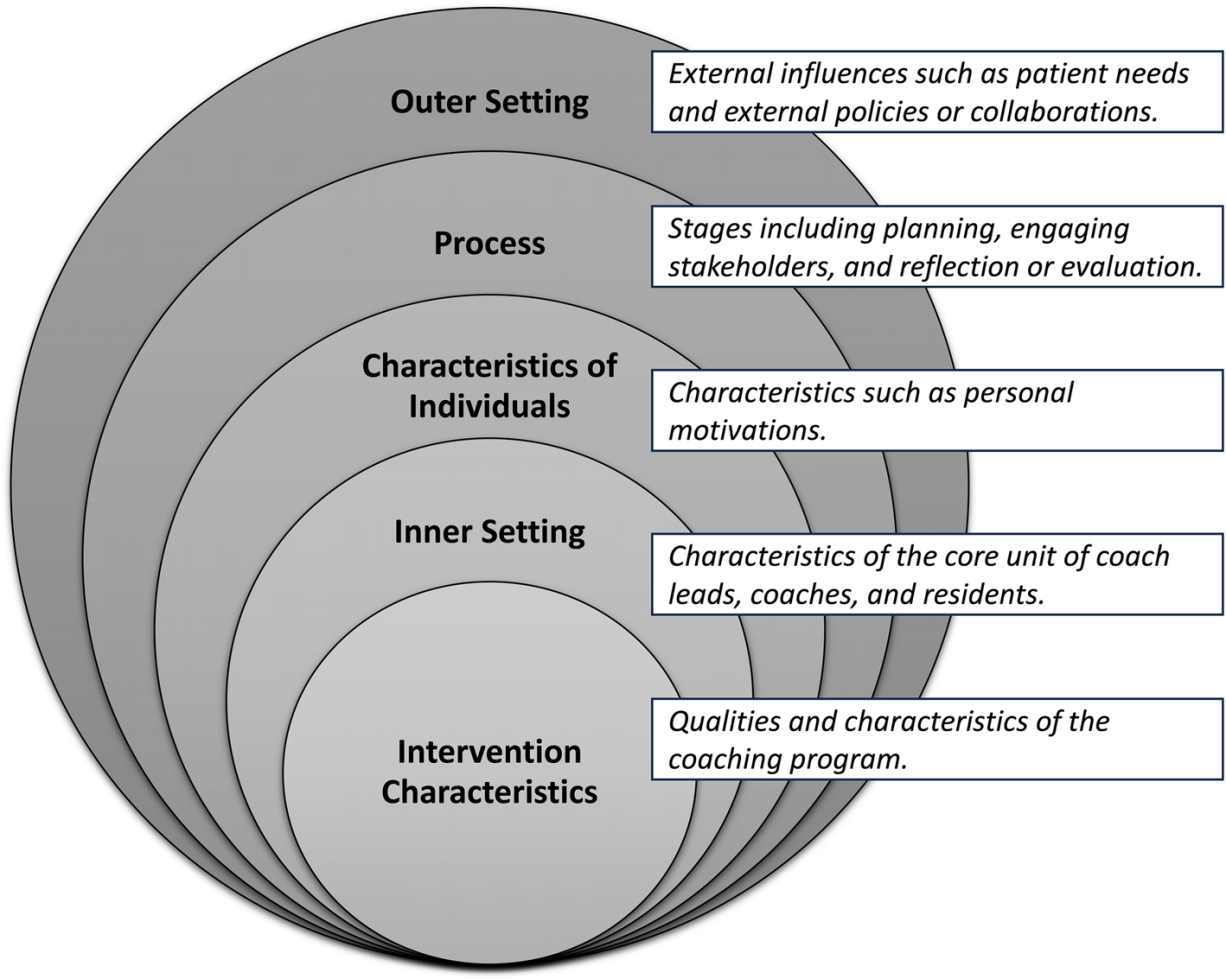
Five Domains Within the CFIR Framework . Note. Figure 1 depicts the 5 domains within the Consolidated Framework of Implementation Research (CFIR): outer setting, inner setting, process, intervention characteristics, and characteristics of individuals. A brief description of each domain within the context of the coaching program is also included

Stanford University implemented a faculty-led communication-focused coaching program in the Departments of Surgery and Neurology in 2020 as previously described [3]. Briefly, the program was designed using Kern’s 6-step model of curriculum development and utilized the Consolidated Framework for Implementation Research in Formative Evaluation (CFIR) as an implementation framework [14, 15]. The CFIR framework breaks down the implementation into 5 domains; see Fig. 1 for the key domains and a description of each. Notably, the program was designed with input from multiple stakeholder groups, including resident coachees, faculty coaches, medical education leaders, and programmatic sponsors, as well as collaborative efforts across multiple levels to ensure a rigorous plan for programmatic evaluation.

### Participants and oversight

We employed a key informant sampling strategy to purposively select study participants, including resident coachees, faculty coaches, medical education leaders, and programmatic sponsors [16]. These four diverse stakeholder groups were, in various roles and functions, involved in the program’s design and implementation.

Our sampling approach aimed for a balanced representation across groups to ensure heterogeneity and capture diverse perspectives [16]. To achieve this, we invited all faculty coaches, medical education leaders, and programmatic sponsors to participate. Additionally, we requested that the Neurology and General Surgery residency chiefs identify approximately a dozen residents from those participating in the program who were willing to join the study. Notably, since the inception of the program in 2020, all Neurology and General Surgery residents in all classes have participated in their respective Communication Coaching Program, totaling well over 200 individuals.

Respondents were recruited via email sent by communication coaching directors (C.A.G. and A.K.N.) or a research analyst (M.S). Each prospective participant received up to three contacts: an initial email and two follow-ups spaced seven days apart. The response rate was high, with all invited faculty coaches, medical education leaders, and programmatic sponsors agreeing to participate. Among residents, only a few declined or failed to respond. Verbal consent was obtained from all participants, and the study was exempt from the IRB review as a quality improvement project.

### Semi-structured interviews

Interview questions were designed using CFIR concepts, with input from experienced medical education and evaluation researchers. They were then pilot-tested with three individuals familiar with the subject but not directly involved in the communication coaching program. Semi-structured interviews were conducted between November 2021 and April 2022 and aimed to capture stakeholders' perspectives on physicians' communication training needs, stakeholders perceived and actual roles, stakeholders’ involvement in the program, factors influencing the implementation process, and strategies for programmatic improvement, sustainment, and spread. The interviews were conducted by a research analyst experienced in qualitative methods (M.S.), lasted 30–45 min, and took place via the Zoom (Zoom Video Communications Inc.) videoconference platform. Interviews were recorded, transcribed verbatim, and deidentified for analysis.

### Analytic approach

A rigorous team-based thematic analysis of the interview transcripts was conducted involving the following six steps: (1) familiarization with the data, (2) generating initial codes, (3) searching for themes, (4) reviewing themes, (5) defining and naming themes, and (6) producing the report [17]. Analytical procedures, including coding and assessing inter-coder agreement, were performed using NVivo qualitative software (Version Pro Enterprise, QSR International Pty Ltd, Massachusetts, USA, 2020). Trustworthiness during each phase of thematic analysis was established by various means, including prolonged engagement with data, peer debriefing, researcher triangulation, use of coding framework, themes and subthemes vetted by team members, team consensus on themes, and thick description of the context [18]

To develop a codebook, three team members (R.M.J., M.S., and U.T.M.) first inductively coded four interviews and then met multiple times to discuss emerging patterns, meanings, and how they fit into the CFIR framework. Then, R.M.J. deductively coded the same set of four interviews, using CFIR constructs as codes, and developed the first draft of the codebook. Next, M.S. and U.T.M. validated the codebook by applying it when independently coding the same four interviews. During this intensive analytical phase, coders frequently met to compare coding, discuss ambiguities, and make adaptions based on the findings, which resulted in the development of a 20-item CFIR-informed codebook. The codebook was then vetted by the whole analytics team (A.K.N., R.K.M., C.A.G., J.R.K., and A.M.M.). After the strong inter-rater agreement was reached (Cohen’s kappa coefficient *κ* = 0.83) between M.S., R.M.J, and U.T.M for the three newly coded interviews, which ensured coding consistency, the coders divided transcripts and coded the remaining interviews, with each coded 11–12 interviews applying the final 20-item codebook [19]. No new codes were identified from this point forward. Throughout the coding and interpretation phases, coders frequently engaged in discussions to identify emerging themes and resolve disagreements. The entire team subsequently reviewed and verified these findings.

The themes that emerged from the CFIR-informed qualitative analysis, were then mapped onto four critical domains of a standard logic model: Context, Inputs and Outputs, Outcomes, and Evaluation. The Context domain describes contextual factors, priorities, and the program landscape as key features of implementation and evaluation. The Inputs and Outputs domain describes the resources invested in the program such as funding, time, skills, technology, and facilities, as well as the personal investment of program personnel and their individual motivation or incentive to engage in the program. This domain also includes the program's content or activities, including program execution and participation. The Outcomes domain addresses the perceived program outcomes and impact. The Evaluation domain focuses on the specifics of programmatic evaluation [12, 13].

### Reflexivity statement

Our research team comprised surgeon-researchers (RMJ, JRK, AMM), neurologist-researchers (RKMK, CAG), and social science researchers (MS, UTM), experienced in qualitative methods, program development, and medical education. Several co-authors (JRK, RKMK, AKN, CAG) had direct involvement in the Communication Coaching program as faculty coaches, medical education leaders, and programmatic sponsors, which provided valuable insider perspectives but also required consideration to minimize potential bias. We adopted a pragmatic approach to study design and analysis, balancing methodological rigor with practical considerations. Our diverse disciplinary backgrounds and roles within the program influenced how we framed research questions, interpreted data, and contextualized findings. To enhance credibility, we engaged in regular discussions to critically examine our assumptions, used multiple perspectives in data interpretation, pilot-tested interview questions with individuals outside the program, and applied the CFIR framework to provide a structured and systematic approach during the entire study.

## Results

### Participant characteristics

Thirty-five stakeholders, including 10 resident coachees (received coaching), 10 faculty coaches (served as coaches and underwent coaching-specific faculty development), 9 medical education leaders (designed and implemented program), and programmatic sponsors (provided financial support), participated in the interviews. Respondents were mainly employees and trainees of the Department of Neurology and Neurological Sciences (16) and the Department of Surgery (16) at Stanford; however, as shown in Table 1, we also interviewed stakeholders from the Stanford Department of Pediatrics, Stanford School of Medicine, and Stanford Healthcare.

**Table 1:** Participant characteristics (*n* = 35)

**Participants**	**Department**			*Total*
	**Neurology**	**Surgery**	**Other**^**a**^	
Resident coachees	6	4	0	*10*
Faculty coaches	4	6	0	*10*
Medical education leaders	4	5	0	*9*
Programmatic sponsors	2	1	5	*8*
*Total*	*16*	*16*	*5*	*37*^*b*^

^a^faculty leaders were from the Stanford Department of Pediatrics (2), Stanford School of Medicine (1), and Stanford Health Care (2)

^b^one participant held various roles (faculty coach, medical education leader, and programmatic sponsor); therefore, the total in the table is greater than the total number of participants

### Key themes and conceptual frameworks utilized

**Fig. 2:**
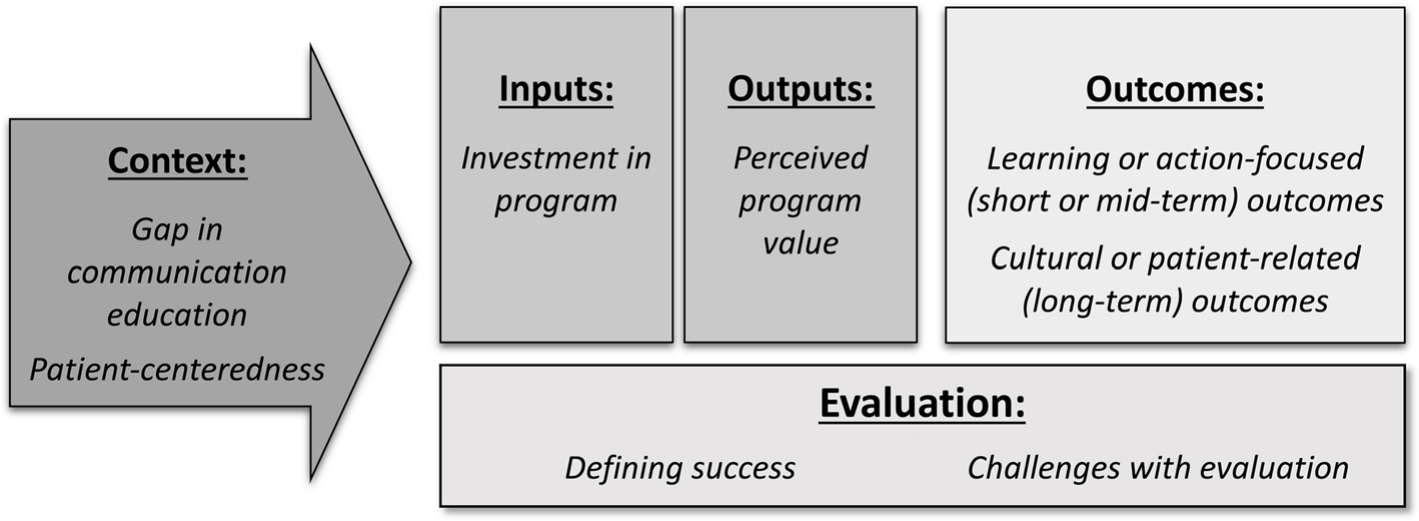
Key Themes Mapped onto a Logic Model for Coaching Program Evaluation. Note. Figure 2 illustrates each of the key themes (italics) identified in the qualitative analysis, mapped onto a distinct domain of a logic model of program evaluation

Eight key themes were identified during the analysis. Figure 2 presents how those CFIR-informed themes were mapped onto the logic model. Table 2 includes illustrated quotations and the CFIR domains from which individual quotations were coded, along with their relationships to each of the logic model domains.

**Table 2:** Representative Quotations and Associated CFIR Domains for Key Themes

Logic Model Domain	Theme	Representative Quotations	Associated CFIR Domains
**Context**	Gap in communication education	- *Learning communication skills was built into a lot of the rotations that we had… Just seeing physicians who are higher than me modeling family meetings… was… how I was able to learn to do it.* (P3 – Coach Neurology) - *It’s typically an unstructured hidden curriculum.* (P12 – Medical Education Leader, Programmatic Sponsor Surgery)	Intervention Characteristics
	Patient-centeredness	- *Patients are more satisfied… There’s an alliance that is formed when there is good communication. I think that not only are patients happier, but they get better care, and they are healthier, probably.* (P4 – Coach Neurology) - *There is a lot of therapeutic benefit to just having good rapport and communication with people.* (P34 – Resident Surgery)	Intervention Characteristics
**Inputs and Outputs**	Investment in program	- *You’ve got to have some… faculty development so that the coaches don’t feel like the blind leading the blind.* (P2 – Coach Neurology) *Funding is extremely important. That comes from buy-in from the hospital, or programs, or chairs, because what makes the coaching program disseminate and sustain itself is a robust reimbursement structure* - (P11 – Medical Education Leader Surgery)	Intervention Characteristics, Inner setting, Characteristics of Individuals, Process
	Perceived program value	• *If it’s tailored to what [the residents] feel is important, then there will… be more ****buy in and engagement****, and that could differ by what year the person is, maybe what specialty they’re in.* (P26 – Resident Neurology) • *There’s a huge value in the relationship building between trainees and faculty, which, I think is confidence building for trainees. It helps trainees… feel more established within the program in general and that they have a safety net in some ways.* (P5 – Coach Surgery)	Intervention Characteristics, Inner Setting
**Outcomes**	Learning or action-focused outcomes	• *Some of the material that we go through, that’s part of the coaching side of the material, is helpful in my own life, in my own clinic… I become a better communicator because of that.* (P1 – Coach Neurology) • *I think a lot of things I kind of just do… We’re very busy as residents, but I think it makes me… in line with self-reflection… it makes me more cognizant of the way I’m doing things, the way I’m phrasing things.* (P34 – Resident Surgery)	Characteristics of Individuals, Process
	Cultural or patient-related outcomes	• *It helps me provide better patient care if I’m better able to communicate with my patients. Obviously, if I am able to communicate more effectively with other teams, that also procures better care for the patients on my team.* (P33 – Resident Surgery) • *It changed the way that we approach feedback in the department, so that our learners are setting their own goals, they’re asking, they’re seeking feedback and it changed our culture from being a performance culture to being much more of a growth mindset. That has extended beyond our residents. I think part of it is just embracing that it can really revolutionize the feedback culture.* (P19 – Programmatic Sponsor Pediatrics)	Inner Setting and Outer Setting
**Evaluation**	Defining success	• *The most successful outcome is improving patients’ satisfaction scores… that’s the ultimate outcome.* (P11 – Medical Education Leader Surgery) • *I think, for myself, if seven years from now, by the time I’m graduating, I sense a change in the surgical culture, where people are notably nurturing each other, helping each other thrive, respectful and happy to be at work together, and are saying things where you can clearly see they’re inspired by each other, I think that would be a success.* (P35 – Resident Surgery)	Process
	Challenges with evaluation	• *It’s really difficult to get great outcomes data for a program like this in the sense of are our residents better communicators today than they were a year ago? That’s difficult to say… But I do know that the residents are happy with the program and on the program side, we certainly get a lot of good written feedback from the communication coaches that give a lot of insight.* (P14 – Medical Education Leader Neurology) • *…one of the holy grails is patient level metrics… but those are also very noisy metrics that have so many different things competing for their input that the signal to noise ratio could be really hard to separate…* (P10 – Medical Education Leader Neurology)	Process

#### Domain 1: Context

The themes* “*Gap in communication education*”* and “ Patient-centeredness*”*, which were mapped onto the Context domain of the logic model, emerged from the CFIR domain of process.

#### Gap in communication education

Participants reported that in their experiences with medical education, communication was infrequently taught or evaluated in a formal setting. Several participants described learning communication through direct or indirect observation rather than in a planned and explicit manner. The few who reported attending classes or receiving specific instruction focused on communication skills typically described a group setting without opportunities for individualized feedback. Thus, this communication coaching program was seen by interviewed stakeholders as a novel means of addressing an unmet need in medical education.

#### Patient-centeredness

Respondents emphasized the importance of patient needs as a critical motivator in improving their own communication and that of other healthcare providers. They suggested that communication was central to a healthy patient-physician relationship. Participants also highlighted the potential for enhanced communication to improve patient care through better patient-provider alliance formation, which could lead to secondary benefits in patient comprehension and adherence to recommended care.

#### Domain 2: Inputs and outputs

The themes *“*Investment in program*”* and *“*Perceived program value*”*, which were mapped onto the Inputs and Outputs domain of the logic model, emerged from multiple CFIR domains, including intervention characteristics, inner setting, characteristics of individuals, and process.

#### Investment in program

Two subthemes were identified within this theme: (1) resource investment and (2) personal investment. For resource investment, our participants highlighted critical resources that were invested across multiple different layers of the program, including education, stakeholder engagement, financial support, and collaboration. For example, faculty development was utilized up-front to help prepare faculty coaches through education about coaching, providing feedback, and facilitating self-reflection. Participants also described the importance of thoughtfully engaging stakeholder groups at the planning stages when making decisions related to program resources and engagement strategies; for instance, residents were included in the interview process for selecting coaches. Financial support from and collaboration with programmatic sponsors was also described as an essential element of program success. Additionally, collaborative efforts such as the mentorship from the Department of Pediatrics, which had previously implemented a coaching program, and the collaboration with Stanford-Surgery Policy Improvement Research & Education Center for an upfront approach to program evaluation were recognized as critical investments from external sources. For personal investment, participants described the individual investment and motivations of program participants, particularly among the program’s leadership team. Their dedication to the coaching effort was thought to be critical to success, and they were frequently described as program champions because of their strong personal commitment to both communication and coaching. Personal motivations to participate were highly variable, but many participants highlighted the importance of communication as an under-addressed skill, an interest in getting more involved in teaching, or a desire for stronger resident/faculty relationships.

#### Perceived program value

Study participants referenced value perceptions across a continuum. Many respondents felt that addressing communication skills through an individualized coaching approach was an important adjunct to existing medical education strategies. They described having a dedicated communication coach as a uniquely valuable element of the program. Many also saw value in the program beyond the benefits to communication, highlighting particularly the value of relationship building between coach and coachee. By contrast, other participants indicated that despite the importance of developing communication skills, the rigid program structure and contrived nature of the program limited its impact and prevented thoughtful engagement. Some participants described time limitations during residency training and highlighted this as a primary challenge to effective engagement, hindering the opportunity to benefit from the program.

#### Domain 3: Outcomes

The themes *“Learning or action-focused (short or mid-term) outcomes”* and *“Cultural or patient-related (long-term) outcomes”*, which were mapped onto the Outcomes domain of the logic model, emerged from the CFIR domains of characteristics of individuals, process, inner setting, and outer setting.

#### Learning or action-focused (short or mid-term) outcomes

Faculty coaches and resident coachees described changes in their own communication-specific behaviors with patients and colleagues as a direct result of the learning that had taken place over the course of the program. Change took the form of increased awareness of their own challenges or limitations related to patient communication, and increased use of and comfort with communication frameworks to guide difficult patient conversations. Participants also recognized behavior change in their interpersonal interactions within healthcare teams and with their coaches/coachees.

#### Cultural or patient-related (long-term outcomes)

Respondents also referenced long-term outcomes, either observed or expected, including changes in culture and improved patient outcomes. Positive culture change was highlighted in multiple areas, including developing a healthier culture of feedback and creating a more nurturing environment at the department level.

#### Domain 4: Evaluation

The themes *“*Defining success*”* and “ Challenges with evaluation*”*, which were mapped onto the Evaluation domain of the logic model, emerged from the CFIR domain of process.

#### Defining success

Study participants described a wide array of potential definitions of programmatic success. While many respondents felt that patient-level data should be considered the “gold standard” of success, others suggested that success could also be measured by resident graduation readiness, faculty-specific metrics related to coaching program utilization, and even perceived department and institutional culture change.

#### Challenges with evaluation

Despite having a clear vision for a successful communication coaching program, participants also described a variety of challenges related to how to effectively measure success within this context. For example, participants perceived difficulty with obtaining outcomes-level data for a communication coaching program. They described challenges associated with using resident milestone evaluations for specific communication encounters. Additionally, they recognized that while improved patient outcomes would generate the most convincing outcomes data, there is considerable noise associated with patient-level metrics.

## Discussion

Effective programmatic change at the GME level requires deliberate program implementation and rigorous program evaluation. In this study, we identified critical elements of the Stanford Neurology and Surgery Communication Coaching Program, considering program inputs, outputs, outcomes, and evaluation metrics, all within the context of our unique environment and individual stakeholder needs. We considered the implementation and evaluation of the coaching program in parallel by combining a commonly used implementation science framework, CFIR, with a common program evaluation method, the logic model.

While program implementation and evaluation are distinct entities, the two go hand-in-hand and should ultimately build on each other in a cyclical fashion to make programs more effective over time and as community and stakeholder needs change [9]. Mapping the key themes identified in our analysis onto a logic model offered a more holistic description of all critical elements of the intervention and exposed areas where the program may not sufficiently meet implementation goals, and even offered suggestions for improvement. Themes that emerged from only one specific stakeholder group or one portion of the logic model may not present the full story of the program; however, in our study, multiple different perspectives contributed to the comprehensive nature of this evaluation, an essential feature of program evaluation [9].

One of the advantages of using the logic model in this way was its emphasis on the relationship of other domains to the program’s context or environment [13]. Our qualitative findings demonstrated a shared perception of a gap in communication education and an emphasis on the importance of communication from a patient perspective. These themes served as a foundation for program implementation, providing common ground for all stakeholder groups. Our findings were consistent with the known importance of a needs assessment in identifying programmatic priorities and specifically seeking to address the needs of the community [20]. The analysis also demonstrated extensive early program investment in time, funding, resources, and personnel. Although the inputs were robust, the evaluation revealed a wider range of participant experiences related to perceived program value, suggesting key differences in the degree of perceived benefit, engagement, and experience in the program. While the linear nature of a logic model has been cited as one of its limitations, [21] clear links between different elements of the model help illuminate discrepancies. Thus, the inequalities between inputs and outputs highlight a potential area for programmatic improvement to better align participant experiences with program objectives and inputs.

**Fig. 3:**
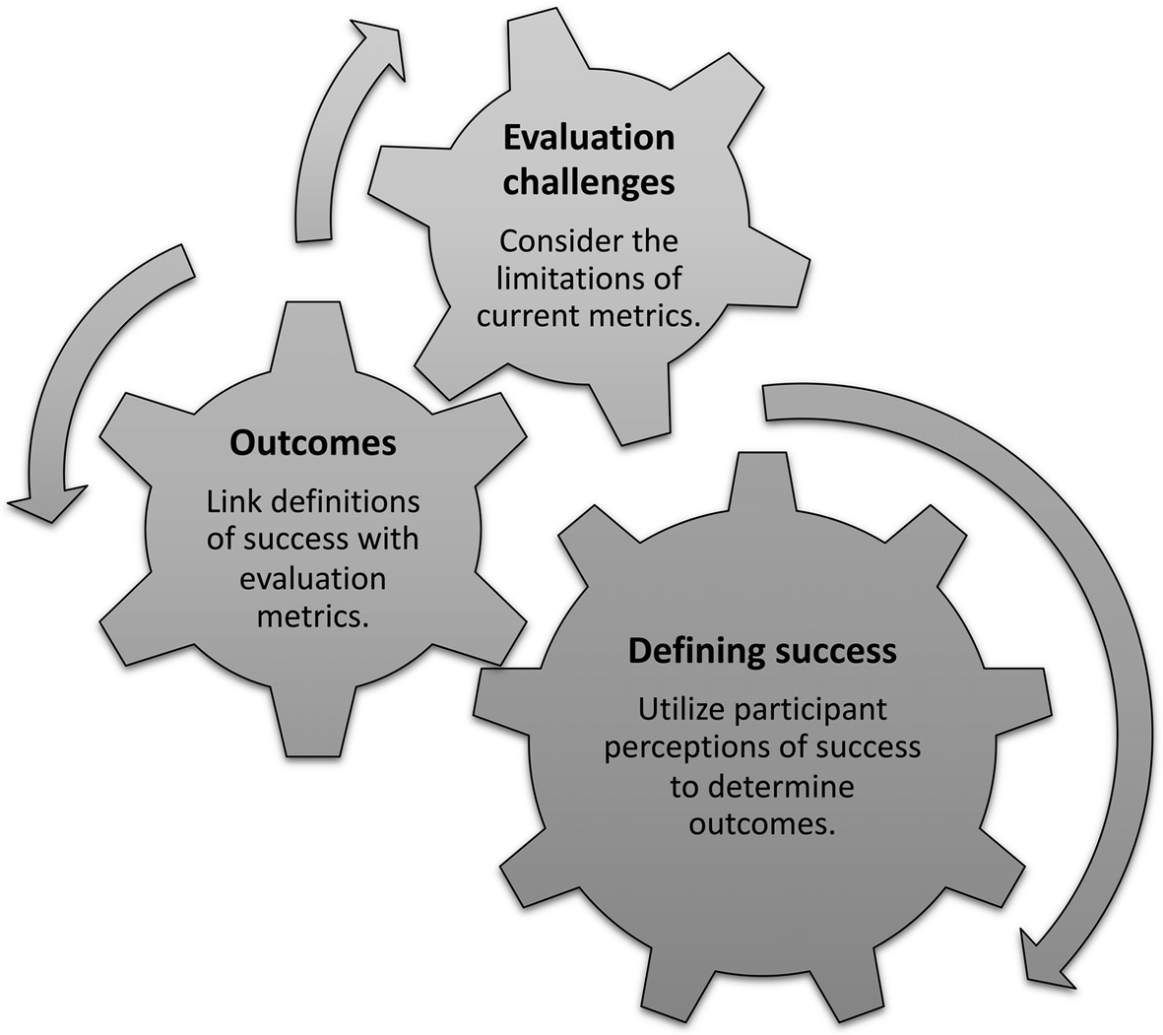
Framework for Success, Outcomes, and Challenges with Evaluation. Note. Figure 3 depicts the complex interplay between definitions of success, outcomes being measured, and evaluation challenges given the limitations of current outcomes metrics

The findings of our study also exposed a unique interplay between definitions of program success, outcomes, and challenges with evaluation. The highly varied descriptions of program success suggested distinct perceptions and experiences both by individual and stakeholder groups. This also introduced potential unintended or unexpected consequences of the program, which are essential to consider in any program evaluation [13]. Although the foundation for the program was firmly rooted in patient-centeredness and a gap in communication education, program participants described successful outcomes much more broadly – at the level of the patient, the resident, the faculty, and even the culture of the institution. We found that participants recognized outcomes and evaluation strategies at multiple Kirkpatrick levels and for various stakeholder groups (i.e., resident perceptions at Level 1, knowledge of communication strategies at Level 2, better non-coach faculty utilization of the coaching program at Level 3, and patient outcomes at Level 4) [22]. Stakeholders also recognized challenges in the measurement of success according to established metrics, such as patient satisfaction scores and resident milestones. These findings ultimately informed a framework from which to consider interwoven concepts of program success, outcomes, and challenges with evaluation to align in medical education interventions (see Fig. 3).

The challenges with existing mechanisms of evaluation further exposed the invaluable nature of the qualitative approach to participant-described outcomes. While it is understandably challenging to see an observed change in patient-level outcomes data, for instance, it brings depth and meaning to the program when participants describe their experience with change, such as the way the intervention has impacted their patient-level communication or interactions with their peers. The perceived definitions of success also indicate that there is room to consider other types of program evaluation metrics, such as perceptions of non-coach faculty, feedback culture, and other patient-level data.

There are several study limitations that warrant further discussion. While 35 separate interviews were conducted, it is possible that some concepts and themes were not represented in this cohort or that findings may be specific to our institution. Participants also had varying degrees of involvement in the program; thus, their experiences may be specific to only some domains of the logic model. However, within each group of interview participants, the researchers felt that thematic saturation was adequately achieved. As well, we believe that our comprehensive program evaluation would be incomplete without input from key stakeholders across departments who can speak to different aspects of the program and various domains of the logic model. Additionally, while patients are a key stakeholder group in the program implementation, they were not included in the qualitative study. Patients have varying levels of contact with the coaching program and many interactions beyond those directly involved in the program. Thus, we determined that it would be too difficult to parse out the impact of the communication coaching program at the level of individual patients.

## Conclusions

In conclusion, the mapping of key themes from this qualitative program evaluation onto the logic model allowed for a holistic review of the distinct yet related elements of the Stanford Neurology and Surgery Residency Communication Coaching Program. Our project has facilitated an iterative process of adjusting the program implementation efforts based on program evaluation findings. We have found this methodology to be a coherent way of linking different programmatic elements to expose strengths and areas for improvement, as well as highlight and measure intended and unintended program outcomes. We will continue to use this strategy to guide future program modifications to meet the changing needs and priorities of stakeholders. A similar methodology should be considered to link implementation and evaluation efforts for coaching programs beyond our institution and for other medical education programs at large.

## Data Availability

The study’s data are stored securely through Stanford University. The data supporting this study’s findings are not publicly available to protect participant identity. However, upon reasonable request, deidentified data are available from the corresponding author.

## Abbreviations

CFIR: The Consolidated Framework of Implementation Research
GME: Graduate Medical Education
